# Quantum dots in axillary lymph node mapping: Biodistribution study in healthy mice

**DOI:** 10.1186/1471-2407-8-111

**Published:** 2008-04-22

**Authors:** Anne Robe, Emilie Pic, Henri-Pierre Lassalle, Lina Bezdetnaya, François Guillemin, Frédéric Marchal

**Affiliations:** 1 CRAN, Nancy-University, CNRS, Centre Alexis Vautrin, Avenue de Bourgogne, 54511 Vandoeuvre-lès-Nancy Cedex, France

## Abstract

**Background:**

Breast cancer is the first cause of cancer death among women and its incidence doubled in the last two decades. Several approaches for the treatment of these cancers have been developed. The axillary lymph node dissection (ALND) leads to numerous morbidity complications and is now advantageously replaced by the dissection and the biopsy of the sentinel lymph node. Although this approach has strong advantages, it has its own limitations which are manipulation of radioactive products and possible anaphylactic reactions to the dye. As recently proposed, these limitations could in principle be by-passed if semiconductor nanoparticles (quantum dots or QDs) were used as fluorescent contrast agents for the *in vivo *imaging of SLN. QDs are fluorescent nanoparticles with unique optical properties like strong resistance to photobleaching, size dependent emission wavelength, large molar extinction coefficient, and good quantum yield.

**Methods:**

CdSe/ZnS core/shell QDs emitting around 655 nm were used in our studies. 20 μL of 1 μM (20 pmol) QDs solution were injected subcutaneously in the anterior paw of healthy nude mice and the axillary lymph node (ALN) was identified visually after injection of a blue dye. *In vivo *fluorescence spectroscopy was performed on ALN before the mice were sacrificed at 5, 15, 30, 60 min and 24 h after QDs injection. ALN and all other organs were removed, cryosectioned and observed in fluorescence microscopy. The organs were then chemically made soluble to extract QDs. Plasmatic, urinary and fecal fluorescence levels were measured.

**Results:**

QDs were detected in ALN as soon as 5 min and up to 24 h after the injection. The maximum amount of QDs in the ALN was detected 60 min after the injection and corresponds to 2.42% of the injected dose. Most of the injected QDs remained at the injection site. No QDs were detected in other tissues, plasma, urine and feces.

**Conclusion:**

Effective and rapid (few minutes) detection of sentinel lymph node using fluorescent imaging of quantum dots was demonstrated. This work was done using very low doses of injected QDs and the detection was done using a minimally invasive method.

## Background

Breast cancer is the first cause of cancer death with a 100% rising in incidence from 1980 to 2000 and 35% growth in mortality [1]. Axillary lymph node dissection (ALND) is the standard surgical treatment in breast cancer patients. It consists in whole lymphatic axillary chain removal with the aim to reveal presence of metastases by histology analyses. However, this procedure leads to numerous morbidity problems such as pain, lymphocel, restriction of arm motion, chronic lymphedema, paresthesia, and, as a consequence, deterioration of life quality. For these reasons, an alternative treatment consisting in the sentinel lymph node (SLN) biopsy has been met with enthusiasm by clinicians. The SLN is the first node in the lymphatic basin into which drains the primary tumour [2]. As a result, axillary lymph node status is the most important prognostic factor and a determinant in the choice of therapy [3]. SLN biopsy consists in ^99m^Tc-radiolabeled sulfur colloid injection the day before surgery in peritumoral or periareolar area [4] followed by lymphoscintigraphy to identify radioactive lymph node(s). During the surgery, a blue dye is injected in the same way and SLN is thus identified as blue and hot lymph node, one of these criterions only is not sufficient for a successful SLN identification [5]. Despite the significant improvement of SLN biopsy over ALDN, the problems related to potential radiation hazards along with an extra cost of the radioisotope, to allergic reactions after blue dye injection and the overall procedure duration are limiting factors for this technique [6]. The ideal method should be sensitive, accurate, rapid, non invasive, non radioactive, and potentially usable in a laparoscopic setting for gastrointestinal tumors [7]. None of the current methods fulfills all of these criteria. Lymphatic mapping with blue dye results in a high rate of false-positive nodes because the small dye particles can readily diffuse through the true SLN and traverse multiple nodes [8]. Additionally, blue dye has poor tissue contrast and is difficult to detect in deep, dark anatomical regions such as the abdomen. Although the use of radioisotope tracers has improved the detection rate and accuracy of SLN mapping, the high radioactivity of the primary injection site can interfere with intraoperative in vivo detection of nearby nodes [3]. Additionally, if radioisotopes are injected during surgery, the time period required for the tracer to migrate to the SLN may delay the operative procedure. Conversely, preoperative injection of the radiocolloid tracer often necessitates anywhere from 2 to 16 hours before surgery. This is difficult, if not impossible, to accomplish and is applicable only in endoscopically accessible regions of the colon and stomach [7].

Quantum dots (QDs) are fluorescent inorganic nanometer sized crystals with remarkable unique optical and electronic properties. They are composed of a fluorescent core of semiconductor heavy metals. In order to limit their potentially toxic effects due to the release of Cd^2+ ^or Se^2- ^ions in case of oxidation of the core (by ultraviolet (UV) light or oxygen) and to passivate the defects on its surface, this core is encapsulated in a shell of organic material, polymeric or lipid-based layers then coat the shell. They have a good quantum yield and absorption cross-section such that single QD fluorescence imaging is possible in cells. QDs have been already applied to visualize SLN in gastrointestinal tract [7], pleural space [9], lung area [10], oesophageal area [11], skin [12] and axilla [13] using infrared light. Recently, red and infra-red emitting QDs were imaged in inguinal and axillary lymph nodes in tumor bearing mice already 3 min after local injection [14]. Major difficulty in using QDs *in vivo *is their unknown potential toxicity. However, it has been demonstrated that the injection of 2.10^9 ^QDs/cell in *xenopus *embryos unaltered their phenotype and health [15] and that QDs triggered apoptosis via reactive oxygen species (ROS) production only when they are not coated with ZnS [16,17]. QDs toxicity study is ultimately related to the investigation of their biodistribution in pre-clinical models. Currently, only few detailed works on QDs biodistribution in rodents have been realized [18-20].

The primary objective of this work was to detect and quantify at different times after the injection the selective accumulation of carboxyl coated QDs in axillary draining lymph nodes. Secondly, we assessed the biodistribution of QDs in different tissues by fluorescence microscopy and chemical extraction procedures. The rationale for the choice of carboxyl coating was based on the observation that negative charge confine to QDs certain selectivity to reticuloendothelial system (RES) [21] along with the excellent retention in lymph nodes [22]. Red emitting 655 nm QDs were considered in this study because of their better fluorescence quantum yield compared to that of IR QDs (fluorescence quantum yield) allowing better quantification with regard to detection level of spectroscopic tools.

## Methods

### Animals

Eight to twelve weeks old female athymic Fox nude mice (Hsd:Athymic Nude-*Foxn1nu*) (Harlan, Gannat, France) weighing from 15 to 25 g were used in these experiments. Mice were kept in 12 h light/dark cycle and had access to food and water *ad libitum *until 24 h before the experimentation. Starvation was performed 24 h before manipulation because of possible food fluorescence interference at the emission wavelength of the QDs. Animal procedures were performed in compliance with institutional and national guidelines. Six groups of animals, each composed of 5 mice, were used for lymphotropism and biodistribution studies: one control group who received only phosphate buffer saline (PBS), and five experimental groups injected with QDs. All experiments were performed under anesthetic using intraperitoneal injection of 0.01 mL/g of body weight of a solution containing 8 mg/mL ketamine (Ketalar^®^, Panpharma, Fougères, France) and 0.8 mg/mL xylazine (Rompun^®^, Bayer Pharma, Puteaux, France).

### Quantum Dots and administration

Quantum Dots (QDs) with a maximum emission peaks at 655 nm were purchased from Quantum Dot Corporation (Qdot^® ^655 ITK™ carboxyl). These QDs are described by the manufacturer as CdSe/ZnS core/shell nanoparticles coated with a polymer having an excess of carboxylic acid groups. Their characteristics include : 16 nm diameter, 94% size monodispersity, extinction coefficient of 800.000 M^-1 ^cm^-1 ^at 638 nm, and a fluorescence quantum yield of 80% with a full width at half maximum of 28 nm. Twenty μL of a 1 μM (20 pmol) QDs solution was injected subcutaneously in the distal part of the right anterior paw, together with 20 μL of 1% patent V blue 2.5%^® ^(Guerbet, Roissy CdG, France) as reference method. The paw was kneaded to allow migration of QDs and blue dye. Mice were sacrificed at 5, 15, 30, 60 min and 24 h after injection by cervical dislocation.

### *In vivo *fluorescence spectroscopy

*In situ *quantification of fluorescence was carried out using laser-induced fluorescence (LIF) as described previously [23]. Two optical fibers were held in direct contact with the axillary lymph node (ALN). One fiber was coupled to a krypton laser (410 nm) to ensure excitation. The second fiber was coupled to a spectrograph (USB2000, Ocean Optics, Dunedin, FL, USA) and a CCD captor transferred to a PC for a fluorescence spectrum acquisition. The fluorescence of the rhodamine B (1 μM in water) was used for the calibration of the excitation light intensity. Fluorescence measurements were performed at three different sites: on the skin away from injection site (belly skin), on the LN through the skin and after skin removal *in vivo*. The measurements were performed at 5, 15, 30, 60 min and 24 h after QDs injection. Measurements in control group were performed at 24 h after PBS injection.

### Fluorescence microscopy

After sacrifice, ALN, bladder, belly skin, spleen, kidney, heart, lung, brain, stomach, liver and intestine were removed and frozen at -80°C. Six micron frozen sections of all organs were prepared after embedding in Tissue Tek^® ^(Sakura Finetek, Torrance, CA, USA). Fluorescence was observed under an upright epifluorescence microscope (AX-70 Provis, Olympus, Rungis, France) equipped with a 100 W mercury vapor lamp and a Peltier cooled CCD camera (DP50, Olympus). The filter set consisted of a 400–440 nm band pass excitation filter associated with a 570 nm dichroic mirror and a 590 nm long pass filter. Fluorescence images were recorded using × 40 enlargement.

### Quantitative determination of QDs

Organs were removed at time points indicated above, weighed and made soluble in Solvable^® ^(Perkin-Elmer, Courtaboeuf, France). Solvable^® ^was added proportionally to organ weight (0.5 mL for a weight ≤ 50 mg, 1.0 mL for a weight ≤ 200 mg and 1.5 mL for a weight ≤ 300 mg). Organs were next incubated at 50°C until the samples become soluble. A sample of each organ, except ALN and bladder, was 40 times diluted in PBS and analyzed by spectrofluorometry (Xenius, SAFAS, Monaco). An excitation wavelength of 350 nm was used and the spectra were collected between 600 and 690 nm. In a parallel set of experiments, a calibration curve for each organ was established while adding a known concentration of QDs to tissue of interest and the fluorescence of the resulting mixture was measured. Blood samples were collected through cardiac puncture in heparinized and cooled tubes 5, 15, 30, 60 min and 24 h after QDs injection. Samples were centrifuged at 3000×g for 10 min at 4°C. Plasma was recovered, ten fold diluted and its fluorescence emission under 450 nm excitation was measured using a spectrofluorometer (PerkinElmer LS50B, Courtaboeuf, France). Urine and feces were collected daily during 2 days. Urine was 10 fold diluted, feces were made soluble by Solvable^® ^at 50°C and the fluorescence of both samples was measured by spectrofluorometry (PerkinElmer LS50B, Courtaboeuf, France) using a 350 nm excitation wavelength.

### Statistical analyses

The non-parametric test of Kruskall-Wallis was employed to establish statistical significance. Mann-Whitney's U test was subsequently used to compare two by two the groups for which Kruskall-Wallis test was significant. All analyses were performed on StatView^® ^5.0 software (Abacus concepts, Berkeley, CA, USA) and all results values were expressed by mean ± SEM (standard error of the mean), * for *P *< 0.05 and ** for *P *< 0.01 compared to the control group.

## Results

### Chemical extraction of QDs in axillary lymph node

**Figure 1:**
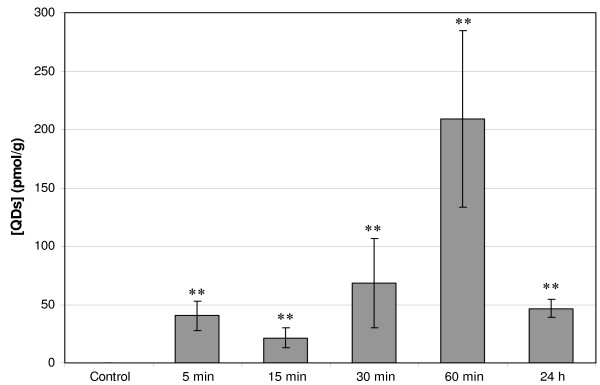
**Concentration of QDs (pmol/g) detected in ALN. **20 pmol were injected subcutaneously in the distal part of the right anterior paw of nude mice. Mice were sacrificed at 5, 15, 30, 60 min and 24 h after injection. Concentration of QDs (pmol/g) was detected in ALN by spectrofluorometry after chemical extraction. Error bars represent SEM, values marked with asterisk were significantly different from control (*P *< 0.01). ALN: Axillary Lymph Node; QDs: Quantum Dots.

Chemical extraction (Figure 1) of QDs in ALN revealed the presence of 40 ± 12 pmol/g tissue in the ALN already 5 min after injection with a maximum of 209 ± 75 pmol/g at 60 min after injection. Approximately a 4 fold fluorescence decrease was observed 24 h post-injection (47 ± 8 pmol/g) (*P *= 0.0088 for all groups). The percentage of migrated QDs (Table 1) has been derived from Figure 1 by calculating the ratio between the quantity of QDs in ALN and the quantity of QDs at the injection (20 pmol). The amount of QDs that migrated to the lymph node 60 min after the injection corresponds to 2.42% of the total injected dose.

**Table 1:** Percentage of QDs injected quantity detected in ALN by spectrofluorometry after chemical extraction.

**5 min**	**15 min**	**30 min**	**60 min**	**24 h**
0.47 ± 0.21% **	0.27 ± 0.10% **	1.00 ± 0.47% **	2.42 ± 0.57% **	1.24 ± 0.34% **

20 pmol were injected subcutaneously in the anterior paw of nude mice. After sacrifice at 5 min, 15 min, 30 min, 60 min and 24 h after injection, ALN was removed, solubilized and the fluorescence of the solution was measured and is expressed as a percentage of the injected doze. Values were expressed by mean ± SEM, values marked with asterisk were significantly different from control (*P *< 0.01). QDs: Quantum Dots; ALN: Axillary Lymph Node

### Fluorescence microscopy experiments

**Figure 2:**
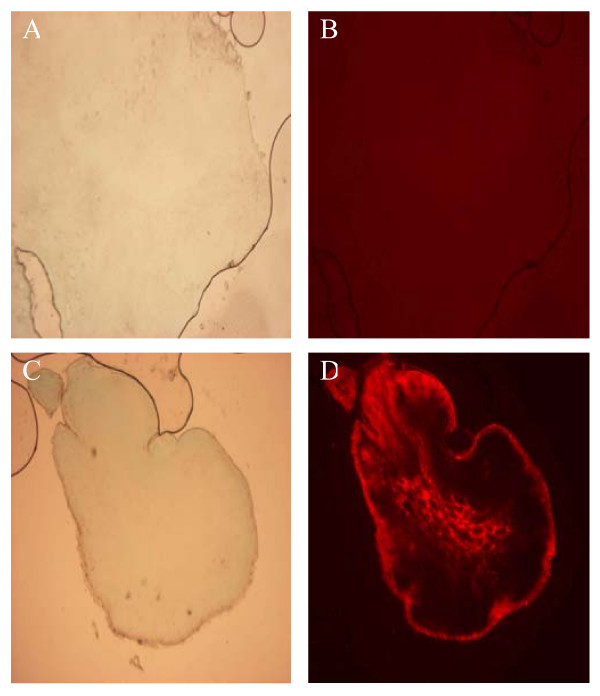
**Transmission and fluorescence microscopy images of ALN frozen sections.** Nude mice were injected with 20 pmol of QDs or PBS (control) in the distal part of the right anterior paw. Panels A, B: transmission and fluorescence images of ALN control; Panels C, D: transmission and fluorescence images of ALN 5 min after QDs injection (40 × enlargement). ALN: Axillary Lymph Node; QDs: Quantum Dots.

Figure 2 displays typical transmission and fluorescence images of lymph nodes frozen sections in control ALN 5 min post-injection. Compared to the background fluorescence, the QDs treated lymph nodes exhibited a distinct fluorescence in the trabecular and medullar sinuses (Figure 2, panel D). It should be noted that QDs fluorescence detected in other examined organs (brain, bladder, spleen, kidneys, heart, lungs, liver and intestine) was not different (data not shown) from the autofluorescence observed in a control lymph node (Figure 2, panel B).

### QDs biodistribution in organs

**Figure 3:**
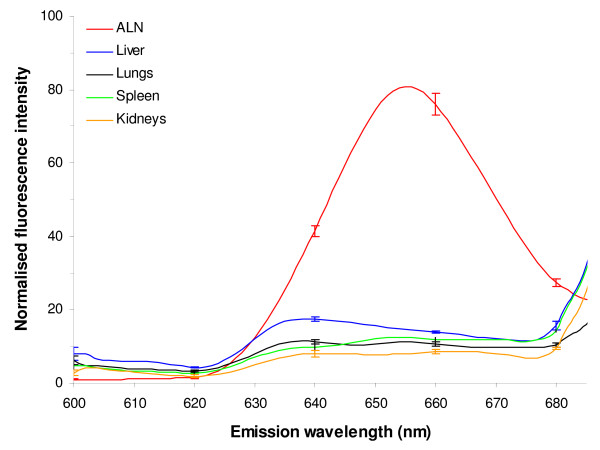
**Normalised fluorescence emission spectra of organs extracts.** ALN, spleen, kidneys, lungs, liver were chemically solubilised 24 h after subcutaneous injection of 20 pmol of QDs. Fluorescence spectra of the extracts were then recorded. Error bars represent SEM. ALN: Axillary Lymph Node; QDs: Quantum Dots.

Mice were injected with QDs, sacrificed 24 h after injection, all organs were removed and subjected to chemical extraction. Figure 3 exhibits typical fluorescence spectra of ALN, liver, lungs, spleen and kidneys. Except for the ALN, no fluorescence peak at 655 nm was detected in the samples. Similar results were found for all time points (5, 15, 30, 60 min and 24 h) and all other tested organs, namely brain, bladder, heart, intestine and skin (data not shown). In a parallel set of experiments we have computed the detection limit of the QDs concentration for each organ using the fluorescence calibration curve. The detection limit ranges from 0.72 pmol of QDs per g of tissue for the bladder to 4.2 pmol/g of tissue for the liver (Table 2). This suggests that the QDs signal in each organ is below the detection thresholds. Extraction of the QDs in the urine and feces was also performed at 48 h post-injection and no fluorescence was detected in the sample pointing out that QDs were not excreted from the body.

**Table 2:** Detection limit of QDs (pmol/g) in each organ after chemical extraction.

**Tissue**	ALN	Liver	Kidneys	Spleen	Lungs	Heart	Intestine	Brain	Bladder	Skin
**Detection limit (pmol/g)**	2.49	4.20	2.20	2.76	2.78	3.98	2.67	2.69	0.72	1.77

QDs: Quantum Dots; ALN: Axillary Lymph Node

### Kinetic of QDs accumulation in lymph nodes by LIF

**Figure 4:**
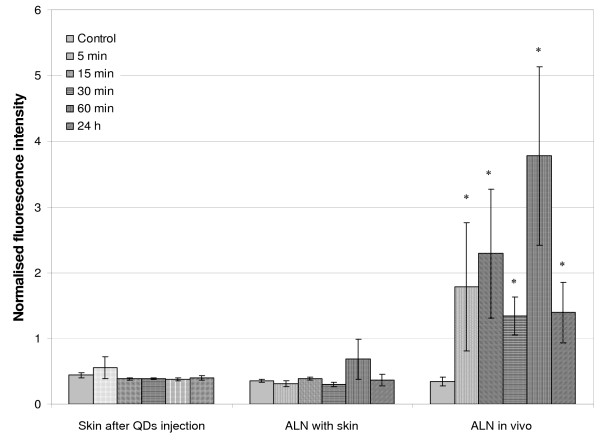
**Normalised 655 nm fluorescence intensity detected by LIF with optical fiber spectrofluorometer.** 20 pmol of QDs or PBS (control mice) were injected subcutaneously in the distal part of the anterior paw of a nude mice. Kinetics of LIF measurements were done on skin after QDs injection, ALN with skin and *in vivo *for control, 5 min, 15 min, 30 min, 60 min and 24 h. Error bars represent SEM, values marked with asterisk were significantly different from control (* for *P *< 0.05). QDs: Quantum Dots; LIF: Laser-Induced Fluorescence; ALN: Axillary Lymph Node.

The *in vivo *kinetics of the QD accumulation in the lymph nodes were studied using fluorescence measurements performed on the animal with a optical fiber spectrofluorometer. No significant fluorescence difference was found concerning the skin aside from the injection point after QDs administration, suggesting that QDs do not migrate into the skin. While QDs emission in the ALN was not detected across the skin, a clear signal was evidenced after incision of the skin and measurement on the ALN (called ALN *in vivo*) (Figure 4) (*P *= 0.0209 for 5, 15, 30 min; *P *= 0.0339 for 60 min and *P *= 0.0143 for 24 h). A very strong fluorescence signal was observed as early as 5 min post-injection (1.79 ± 0.97). *In vivo *measured animals exhibited a persistent fluorescence till 60 min observation (3.78 ± 1.36). Except of 30 min time point, we observed a gradual increase in fluorescence till 60 min. This time point (30 min) is likely suffered from a complexity of optical measurements. A considerable fluorescence decrease was observed at 24 h post-injection (1.40 ± 0.46). Measurements performed at the injection site showed very high intensity peak (*ca*. 4000 a.u.) with no marked difference in intensity during observation time, till 24 h (data not shown). This suggested that the majority of the injected quantity remained at the injection site.

## Discussion

*In vivo *biodistribution studies of QDs are sparse and deal mostly with intravenously (IV) injected QDs. The general consensus is that QDs distribution depends on their physicochemical properties and in particular, size and coating. Tracer particle size has an important effect on migration time in SLN mapping. Particles < 5 nm diffuse into blood, those between 5 to 10 nm can migrate through nodal tissue and result in false-positive results, and those > 1000 nm largely remain at the injection site. Consequently, we choose QDs with a diameter of 16 nm, since a size range between 15 and 20 nm is optimal to enable the QDs to travel through the lymph channels and be trapped by SLN [9,12]. Concerning coating, we choose carboxyl coated QDs. The carboxyl groups present the property to be negatively charged and therefore display rapid uptake in lymphatics and excellent retention in lymph nodes [12,21,24].

To our knowledge, a single biodistribution study was carried out after non-IV administration of QDs. Gopee *et al*. [19] investigated the distribution in healthy mice of 48 pmol intradermally (ID) injected QDs with a diameter of 37 nm and PEG coating. The authors did not find any QDs in lymph nodes until 12 h after injection, whereas we could detect them already 5 minutes following the injection (Figure 1). An early detection (3 min) of QDs traveling toward inguinal nodes was observed after intratumoral injection of red emitting QDs at concentrations similar to ours [14]. However this observation was not followed by biodistribution investigations. QDs Fluorescence increases with time to reach a maximum at 60 min after the injection. The calculated percentages of the injected dose (2.42% at 60 min and 1.24% at 24 h) (Table 1) were comparable with those of Kim *et al*. [13] and Gopee *et al*. [19] who found 2–4% and 1.07% respectively in the SLN. We observed a 4 fold decrease of QDs concentration in ALN from 60 min to 24 h post-injection (Figure 1). We also noted a strong fluorescence of QDs at the injection site. This observation is consistent with the study of Gopee and co-workers [19] who found 60% of cadmium retention at the injection site. We further undertook the study of the biodistribution of QDs in the organs. No fluorescence peak was observed after chemical extraction of Qdot^® ^655 ITK™ carboxyl in liver, kidneys, spleen, lungs, heart, intestine, brain, bladder, and skin (Figure 3). This points out that we are below the calculated concentration limit (Table 2), even if the latter is as low as around 2.5 pmol/g of tissue. The lower detection threshold observed for pigmented organs such as liver and heart was expected (Table 2) since the fluorescence detection method employed in our study is very sensitive to the absorption properties of the sample. In contrast to our results, the previous QDs biodistribution studies all evidenced the presence of QDs in the liver and also to a minor extent in spleen and kidneys [18,19]. The plausible explanation could be provided by the difference in the site of injection. Gopee *et al*. [19] injected QDs ID in the dorsal flank and found 24 h after injection 6.40% of the initial dose in liver and a small amount in kidneys (0.51%) and spleen (0.18%). We have administrated QDs subcutaneously in the paw. ID injection of QDs in the flank drains differently from the SC injected QDs [25] because of the anatomical differences in lymphatic network of the two regions. Likewise, when intradermal injection is performed on the back, direct lymphatic drainage to retroperitoneal and paravertebral nodes can occur before the injected product is distributed throughout the body [26]. When injection is performed in the paw, the first lymph node that receives direct lymphatic drainage is always in the axillary area. QDs are unlikely to be excreted from the body, neither by urine nor by feces in the time span of 48 h. This observation is in agreement with previous work [18] demonstrating the absence of QDs in urine and feces during ten days. Theses results are explained by the dramatic reduction of renal filtration for QDs with a hydrodynamic diameter higher than 3.5 nm and with negative charges [27]. Taken as a whole, the decline in QDs concentration 24 h post-injection could not be attributed to the elimination and/or migration of QDs to different organs. Assuming very strong retention in SLN, we hypothesize that QDs may diffuse in the second lymph node of the chain. This supposition is consistent with the fluorescence distribution pattern of QDs in SLN (Figure 2). Indeed QDs fluorescence is extended till medulla sinus, thus rending possible the migration to the distal lymph node through the lymph channels. The interaction of QDs with serum should also be addressed. We did not perform studies on the interaction of carboxyl QDs with serum proteins, however, as it was shown recently anionic or cationic charged QDs were associated with an increase of the hydrodynamic diameter after incubation with serum [20]. Moreover, the intensity of fluorescence of QDs increases in the presence of BSA [28] or hemoglobin [29].

Spectrofluorometry using an optical fiber is a non-invasive procedure for the detection of fluorescent tracers. The results of our study demonstrate that despite the incapacity to detect QDs directly through the skin, LIF measurements allow the detection of QDs in ALN after skin incision with a profile similar to chemical extraction (Figure 4). However, non-invasive measurements are frequently suffering from significant variations of measurements, related to light diffusion and scattering. Further, actually, our work is a preliminary step to use of NIR emitting QDs which we expect the detection of QDs through the skin without any incision. In the future, the non-invasive measurements could be achieved by modulating an excitation wavelength and using NIR emitting QDs. The excitation wavelength in our study was set to 410 nm. Using an excitation at larger wavelengths should result in less skin absorption and scattering, and should make deep tissue imaging possible. Detection of QDs with optical fiber spectroscopy opens perspectives for the utilization of this technique in operation theatre, since it can provide a rapid and reliable QDs detection immediately after SLN excision. A limitation to the clinical use of QDs for SLN mapping is their potential toxicity [30]. However, because most of the injected dose is eliminated by removal of the injection site and nodal tissue, the eventual toxicity may be negligible. Furthermore, to avoid toxicity caused by biodegradation of QDs and exposition of core metal ions, QDs with non-heavy metal cores (such as indium) would be developed along with ameliorations of surface chemistry [31].

## Conclusion

In this study we demonstrated that 655 nm emitting QDs with carboxyl coating allow the visualization of axillary draining lymph node (ALN) after subcutaneous injection of a very weak quantity of QDs (20 pmol). Unlike other tracers, QDs rapidly localize to and remain trapped in the SLN. A strong fluorescence signal is obtained within a few minutes lasting at least 24 h as measured with optical fiber spectrofluorometry, a minimally invasive technique allowing rapid pre-operative complete procedure. Fluorescence microscopy and chemical extraction confirmed highly selective QDs accumulation in the ALN corresponding to 2.42% of the injected dose at 60 min after the injection; the major part remaining at injection site. This rapid and selective accumulation of QDs in ALN together with the eventual non-invasive fluorescence detection offers an exciting opportunity to track lymphatic flow in real time and guide their nodal dissection after one simple intraoperative injection.

## List of abbreviations

au: arbitrary unit; ALN: Axillary Lymph Node; ALND: Axillary Lymph Node Dissection; ICP-AES: Inductive Coupled Plasma Atomic Emission Spectroscopy; ID: Intradermally; IR: Infra-Red; IV: Intravenously; LIF: Laser-Induced Fluorescence; MUA: Mercaptoundecanoic acid; NIR: Near-infrared; PBS: Phosphate Buffer Saline; QD: Quantum Dots; RES: Reticuloendothelial System; ROS: Reactive Oxygen Species; SC: Subcutaneously; SEM: Standard Error of the Mean; SLN: Sentinel Lymph Node; UV: Ultra-Violet.

## Competing interests

The author(s) declares that they have no competing interests.

## Authors' contributions

AR and EP carried out the experimental and mouse studies. AR and EP participated in the pathological studies and performed the statistical analysis. AR, LB, FG and FM participated in the design and coordination of the study. AR, HPL, LB and FM drafted the manuscript. All authors read and approved the final manuscript.

## Pre-publication history

The pre-publication history for this paper can be accessed here:



## References

[B1] Remontet L, Esteve J, Bouvier AM, Grosclaude P, Launoy G, Menegoz F, Exbrayat C, Tretare B, Carli PM, Guizard AV, Troussard X, Bercelli P, Colonna M, Halna JM, Hedelin G, Mace-Lesec'h J, Peng J, Buemi A, Velten M, Jougla E, Arveux P, Le Bodic L, Michel E, Sauvage M, Schvartz C, Faivre J (2003). Cancer incidence and mortality in France over the period 1978-2000. Rev Epidemiol Sante Publique.

[B2] Jakub JW, Pendas S, Reintgen DS (2003). Current status of sentinel lymph node mapping and biopsy: facts and controversies. Oncologist.

[B3] Marchal F, Rauch P, Morel O, Mayer JC, Olivier P, Leroux A, Verhaeghe JL, Guillemin F (2006). Results of preoperative lymphoscintigraphy for breast cancer are predictive of identification of axillary sentinel lymph nodes. World J Surg.

[B4] D'Eredita G, Giardina C, Guerrieri AM, Berardi T (2006). A further validation of subareolar injection technique for breast sentinel lymph node biopsy. Ann Surg Oncol.

[B5] Wilke LG, McCall LM, Posther KE, Whitworth PW, Reintgen DS, Leitch AM, Gabram SG, Lucci A, Cox CE, Hunt KK, Herndon JE, Giuliano AE (2006). Surgical complications associated with sentinel lymph node biopsy: results from a prospective international cooperative group trial. Ann Surg Oncol.

[B6] Sato K (2007). Current technical overviews of sentinel lymph node biopsy for breast cancer. Breast Cancer.

[B7] Soltesz EG, Kim S, Kim SW, Laurence RG, De Grand AM, Parungo CP, Cohn LH, Bawendi MG, Frangioni JV (2006). Sentinel lymph node mapping of the gastrointestinal tract by using invisible light. Ann Surg Oncol.

[B8] Kaleya RN, Heckman JT, Most M, Zager JS (2005). Lymphatic mapping and sentinel node biopsy: a surgical perspective. Semin Nucl Med.

[B9] Parungo CP, Colson YL, Kim SW, Kim S, Cohn LH, Bawendi MG, Frangioni JV (2005). Sentinel lymph node mapping of the pleural space. Chest.

[B10] Soltesz EG, Kim S, Laurence RG, DeGrand AM, Parungo CP, Dor DM, Cohn LH, Bawendi MG, Frangioni JV, Mihaljevic T (2005). Intraoperative sentinel lymph node mapping of the lung using near-infrared fluorescent quantum dots. Ann Thorac Surg.

[B11] Parungo CP, Ohnishi S, Kim SW, Kim S, Laurence RG, Soltesz EG, Chen FY, Colson YL, Cohn LH, Bawendi MG, Frangioni JV (2005). Intraoperative identification of esophageal sentinel lymph nodes with near-infrared fluorescence imaging. J Thorac Cardiovasc Surg.

[B12] Tanaka E, Choi HS, Fujii H, Bawendi MG, Frangioni JV (2006). Image-guided oncologic surgery using invisible light: completed pre-clinical development for sentinel lymph node mapping. Ann Surg Oncol.

[B13] Kim S, Lim YT, Soltesz EG, De Grand AM, Lee J, Nakayama A, Parker JA, Mihaljevic T, Laurence RG, Dor DM, Cohn LH, Bawendi MG, Frangioni JV (2004). Near-infrared fluorescent type II quantum dots for sentinel lymph node mapping. Nat Biotechnol.

[B14] Ballou B, Ernst LA, Andreko S, Harper T, Fitzpatrick JA, Waggoner AS, Bruchez MP (2007). Sentinel Lymph Node Imaging Using Quantum Dots in Mouse Tumor Models. Bioconjug Chem.

[B15] Dubertret B, Skourides P, Norris DJ, Noireaux V, Brivanlou AH, Libchaber A (2002). In vivo imaging of quantum dots encapsulated in phospholipid micelles. Science.

[B16] Chan WH, Shiao NH, Lu PZ (2006). CdSe quantum dots induce apoptosis in human neuroblastoma cells via mitochondrial-dependent pathways and inhibition of survival signals. Toxicol Lett.

[B17] Cho SJ, Maysinger D, Jain M, Roder B, Hackbarth S, Winnik FM (2007). Long-term exposure to CdTe quantum dots causes functional impairments in live cells. Langmuir.

[B18] Fischer HC, Liu L, Pang KS, Chan WCW (2006). Pharmacokinetics of nanoscale quantum dots: in vivo distribution, sequestration, and clearance in the rat. Advanced Functional Materials.

[B19] Gopee NV, Roberts DW, Webb P, Cozart CR, Siitonen PH, Warbritton AR, Yu WW, Colvin VL, Walker NJ, Howard PC (2007). Migration of Intradermally Injected Quantum Dots to Sentinel Organs in Mice. Toxicol Sci.

[B20] Choi HS, Liu W, Misra P, Tanaka E, Zimmer JP, Itty Ipe B, Bawendi MG, Frangioni JV (2007). Renal clearance of quantum dots. Nat Biotechnol.

[B21] Gao X, Cui Y, Levenson RM, Chung LW, Nie S (2004). In vivo cancer targeting and imaging with semiconductor quantum dots. Nat Biotechnol.

[B22] Frangioni JV, Kim SW, Ohnishi S, Kim S, Bawendi MG (2007). Sentinel Lymph Node Mapping With Type-II Quantum Dots. Methods Mol Biol.

[B23] Kamuhabwa AA, Cosserat-Gerardin I, Didelon J, Notter D, Guillemin F, Roskams T, D'Hallewin MA, Baert L, de Witte PA (2002). Biodistribution of hypericin in orthotopic transitional cell carcinoma bladder tumors: implication for whole bladder wall photodynamic therapy. Int J Cancer.

[B24] Hawley AE, Davis SS, Illum M (1995). Targeting of colloids to lymph nodes: influence of lymphatic physiology and colloidal characteristics. Advanced Drug Delivery Reviews.

[B25] Kersey TW, Van Eyk J, Lannin DR, Chua AN, Tafra L (2001). Comparison of intradermal and subcutaneous injections in lymphatic mapping. J Surg Res.

[B26] Uren RF (2004). Lymphatic drainage of the skin. Ann Surg Oncol.

[B27] Frangioni JV (2003). In vivo near-infrared fluorescence imaging. Curr Opin Chem Biol.

[B28] Yu Y, Lai Y, Zheng X, Wu J, Long Z, Liang C (2007). Synthesis of functionalized CdTe/CdS QDs for spectrofluorimetric detection of BSA. Spectrochim Acta A Mol Biomol Spectrosc.

[B29] Hu DH, Wu HM, Liang JG, Han HY (2007). Study on the interaction between CdSe quantum dots and hemoglobin. Spectrochim Acta A Mol Biomol Spectrosc.

[B30] Lin S, Xie X, Patel MR, Yang YH, Li Z, Cao F, Gheysens O, Zhang Y, Gambhir SS, Rao JH, Wu JC (2007). Quantum dot imaging for embryonic stem cells. BMC Biotechnol.

[B31] Michalet X, Pinaud FF, Bentolila LA, Tsay JM, Doose S, Li JJ, Sundaresan G, Wu AM, Gambhir SS, Weiss S (2005). Quantum dots for live cells, in vivo imaging, and diagnostics. Science.

